# From Guidelines to Real‐Time Conversation: Expert‐Validated Retrieval‐Augmented and Fine‐Tuned GPT‐4 for Hepatitis C Management

**DOI:** 10.1111/liv.70349

**Published:** 2025-09-17

**Authors:** Mauro Giuffrè, Nicola Pugliese, Simone Kresevic, Milos Ajcevic, Francesco Negro, Massimo Puoti, Xavier Forns, Jean‐Michel Pawlotsky, Dennis L. Shung, Alessio Aghemo

**Affiliations:** ^1^ Department of Internal Medicine (Section of Digestive Diseases), Yale School of Medicine Yale University New Haven Connecticut USA; ^2^ Department of Medicine, Surgery and Health Sciences University of Trieste Trieste Italy; ^3^ Department of Biomedical Sciences Humanitas University Milano Italy; ^4^ Division of Internal Medicine and Hepatology, Department of Gastroenterology IRCCS Humanitas Research Hospital Milano Italy; ^5^ Department of Engineering and Architecture University of Trieste Trieste Italy; ^6^ Division of Gastroenterology University Hospitals of Geneva Geneva Switzerland; ^7^ Division of Infectious Diseases ASST GOM Niguarda Milan Italy; ^8^ Department of Medicine University of Milan Bicocca Milan Italy; ^9^ Liver Unit, Hospital Clínic University of Barcelona, IDIBAPS Barcelona Spain; ^10^ Consorcio de Investigación Biomédica en Red de Enfermedades Hepáticas y Digestivas, ISCIII Madrid Spain; ^11^ Université Paris Est Créteil, INSERM, IMRB Créteil France; ^12^ Inserm, U955, Team18 Créteil France; ^13^ Department of Digestive and Hepatobiliary Surgery Assistance Publique‐Hôpitaux de Paris, Paul Brousse University Hospital Paris France

**Keywords:** ChatGPT, generative artificial intelligence, generative pretrained models, hepatitis C virus, large language models

## Abstract

**Background and Aims:**

Advances in artificial intelligence, particularly large language models (LLMs), hold promise for transforming chronic disease management such as Hepatitis C Virus (HCV) infection. This study evaluates the impact of retrieval‐augmented generation (RAG) and supervised fine‐tuning (SFT) on both open‐ended question answering (accuracy and clarity) and on LLM‐recommended treatment regimens for clinical scenarios.

**Methods:**

We employed OpenAI's GPT‐4 Turbo in four configurations—baseline, RAG‐Top1, RAG‐Top 10 and SFT—using the 2020 EASL HCV guidelines as external knowledge or fine‐tuning data. For the question set, guidelines were segmented at the paragraph level and encoded into 3072‐dimensional embeddings. Fifteen questions covering general, patient and physician perspectives were scored on a 10‐point accuracy scale and binary accuracy/clarity by four experts. Separately, we created 25 simulated clinical scenarios; a consensus of four hepatologists defined the gold‐standard DAA regimens. Model performance on these cases was measured by two metrics: ‘partial accuracy’ (≥ one correct DAA without errors) and ‘complete accuracy’ (all correct DAAs without errors).

**Results:**

On open‐ended questions, RAG‐Top10 outperformed baseline in accuracy (91.7% vs. 36.6%; *p* < 0.001) and clarity (91.7% vs. 46.6%; *p* < 0.001). RAG‐Top1 achieved 81.7% accuracy and 86.6% clarity (both *p* < 0.001), while SFT reached 71.7% accuracy and 88.3% clarity (*p* < 0.001). Similarly, RAG‐Top10 achieved the highest performance in prescribing the correct DAA regimen according to expert consensus in 76% of cases (vs. 24% for baseline model, *p* < 0.001).

**Conclusions:**

Both RAG‐Top10 and SFT markedly enhance LLM performance in guideline‐driven HCV management—improving not only response accuracy and clarity but also DAA selection in clinical scenarios. RAG‐Top10's broader context retrieval confers the greatest gains, while SFT underscores the value of domain‐specific alignment. Rigorous, expert‐informed evaluation frameworks are essential for the safe integration of LLMs into clinical practice.

**Summary**

- Advances in large language models (LLMs) research offer the potential for transforming and automating chronic disease management, such as Hepatitis C Virus (HCV) infection. This study evaluates the impact of retrieval augmented generation (RAG) and supervised fine‐tuning (SFT) on the accuracy and clarity of LLM‐generated HCV management recommendations.
- Retrieval strategies that incorporate broader contextual information significantly improved model performance. Models with retrieval‐augmented generation demonstrated higher accuracy (up to 91.7%) and clarity (up to 91.7%) compared to the baseline GPT‐4 Turbo model (36.6% accuracy, 46.6% clarity). Similarly, SFT‐enhanced GPT‐4 improved accuracy (71.7%) and clarity (88.3%).
- Integrating RAG and SFT into LLMs enhances the quality of HCV management recommendations by refining outputs to align with gold‐standard guidelines. These approaches support faster access to medical knowledge in clinical decision‐making, potentially increasing adherence to guidelines and providing precise, understandable advice for patient care.

## 1 Introduction

Large language models (LLMs) represent a novel technology that has the potential to increase the availability of expertise across a variety of industries, including healthcare. LLMs may have particular value in the management of Hepatitis C Virus (HCV) infection, where complex management decisions span the spectrum of clinical activity such as answering medical queries, interactive dialogue systems with patients and understanding clinical reports [1, 2, 3, 4, 5]. The management of chronic HCV infection is a complex challenge for healthcare providers, who must choose the most appropriate treatment strategy from a wide range of options [6, 7, 8]. This decision‐making process typically involves reference to current clinical guidelines to ensure the application of best evidence‐based practices. However, uptake of guideline‐based recommendations for HCV can be suboptimal [9, 10, 11], which may be in part due to decreased ability to integrate guidelines rapidly at the point of care, especially for screening and follow‐up recommendations. LLMs may be helpful for clinical decision support, offering the ability to rapidly process relevant patient information and provide customised treatment or follow‐up recommendations according to established guidelines [12].

LLMs currently demonstrate variable accuracy in digestive diseases, with reported accuracy ranging from 6.4% to 91.4% [13, 14]. Ensuring the accuracy and completeness of the information generated is crucial in clinical practice, where over‐reliance on inaccurate LLM‐generated recommendations could lead to patient harm [15, 16]. Current research in the use of LLMs in digestive diseases is limited by the lack of a widely accepted definition of accuracy and the absence of standardised methods regarding model version and settings, the number and experience of the human evaluators, and the number and type of questions asked. Strategies to increase output accuracy are being explored, using retrieval augmented generation (RAG) or supervised fine‐tuning (SFT) to align foundational LLMs to human expectations [2, 17, 18, 19, 20, 21, 22, 23]. With RAG, the foundational model retrieves information from an external information dataset and binds its response generation to the relevant information contained in the context and the instruction provided by the user through prompt engineering. Fine‐tuning, on the other hand, is the adaptation of a pre‐existing LLM undergoing additional training phases on a more task‐specific labelled (supervised) dataset by changing existing parameters (e.g., weights and biases in the neural network architecture). Integrating RAG allows LLMs to access large amounts of external data, offering real‐time updates and contextually enriched responses [17]. Fine‐tuning can create highly specialised models tailored to specific domains [19, 20, 24]. However, this process can inadvertently narrow the model's breadth of knowledge. It can also be resource‐intensive, requiring continuous updating with new data to prevent the model from becoming outdated, which may limit its practicality in rapidly evolving fields such as medicine. Recent changes to the increased availability of user‐friendly fine‐tuning through the OpenAI application programming interface now allow for exploring these techniques in medical applications. To date, only two studies have investigated the potential of fine‐tuning in digestive disease [21, 25], with few studies using RAG strategies to improve the accuracy of GPT‐4 in predicting adequate follow‐up recommendations after polypectomy, finding that RAG implementation with relevant guidelines improved GPT‐4 accuracy by approximately 30% (from 50.5% to 79%, *p* < 0.01) [26, 27].

The primary outcome of this study is to explore the variation in accuracy and clarity by the implementation of RAG or SFT, with outputs graded by a series of experts among the main authors and chairs of the relevant European Guidelines for HCV management and hepatologists from a tertiary referral centre. We also aim to determine the optimal RAG strategy by comparing different text chunking approaches (sentence‐based, fixed‐length and paragraph‐based) and retrieval configurations (single chunk vs. multiple chunks) to identify the most effective framework for clinical guideline retrieval. Furthermore, we investigate the impact of model hyperparameter tuning, specifically examining how temperature and top‐*p* values affect model performance and response similarity to expert answers. Additionally, we evaluate these optimised models' ability to provide accurate treatment recommendations in clinical scenarios. To this end, we test baseline, RAG and SFT models across 25 simulated HCV clinical cases, assessing their ability to recommend appropriate direct‐acting antiviral (DAA) regimens that align with expert consensus. These comprehensive evaluations will help determine whether augmented LLMs can reliably support clinical decision‐making in HCV management and identify which enhancement approach—RAG or SFT—offers the most substantial improvements in accuracy, clarity and clinical applicability.

## 2 Methods

### 2.1 Question and Clinical Cases Generation

Two junior hepatologists, M.G. (4 years of experience, 100 patients treated/yearly) and N.P. (6 years of experience, 100 HCV patients treated/yearly), selected four principal domains in the guideline (i.e., general indications to treatment, pre‐therapeutic assessment, drug–drug interactions and treatment of HCV patients with renal impairment) and five principal topics within the four domains and drafted representative questions to reflect general, patient‐oriented and physician‐oriented perspectives for each of the topics (Table 1). Each question was generated to specifically target the retrieval of a set of information contained in single or multiple paragraphs of the guideline. Each question was reviewed and approved by an expert hepatologist who contributed as one of the main panel members of the European Association for the Study of the Liver (EASL) HCV guidelines, A.A. (20 years of experience, 100 HCV patients treated/yearly). In addition, the two junior hepatologists (M.G. and N.P.) who drafted the questions also provided free‐text answers, which were reviewed and approved by the senior hepatologist (A.A.). These approved answers were then used as the gold standard text to which LLM responses were compared using various text‐similarity metrics to assess the best chunking strategy and hyperparameters tuning.

**TABLE 1:** List of questions across four domains and three perspectives (general, patient and physician‐oriented) generated by two hepatologists and reviewed by one of the panel members of Hepatitis C Virus Management Guidelines published by the European Association for the Study of the Liver.

**Domain #1: General indications to treatment: who should be treated?**	
General	Is therapy with direct‐acting antivirals recommended for all patients diagnosed with chronic hepatitis C?
Patient	I have been diagnosed with hepatitis C. Do I need to take antiviral therapy even if blood tests and abdominal ultrasound show no signs of liver disease?
Physician	Should I treat a young patient recently diagnosed with chronic hepatitis C with normal transaminases and no fibrosis assessed by non‐invasive methods with direct‐acting antivirals?
**Domain #2: Pretherapeutic assessment: HCV genotype determination**	
General	Is it necessary to know the HCV genotype before starting therapy with direct‐acting antivirals?
Patient	I have been diagnosed with hepatitis C and am about to start antiviral therapy. The doctor recommended viral genotyping, but I did not understand its usefulness. Do I need to have the result before starting antiviral therapy?
Physician	I have recently seen a newly diagnosed HCV patient from a geographical area where HCV subtypes that are intrinsically resistant to NS5A inhibitors are common. Do I need to test him for HCV genotype before starting first‐line antiviral therapy?
**Domain #3: Drug–Drug Interaction Assessment**	
General	Is it possible to start therapy with direct‐acting antivirals in a patient with chronic HCV infection and on anticoagulants therapy?
General	Is it possible to start therapy with direct‐acting antivirals in a patient with chronic HCV infection and on statins?
Patient	This morning the hepatologist prescribed me Sofosbuvir/Velpatasvir for a chronic HCV infection. I forgot to tell him that I'm taking dabigatran for atrial fibrillation. Can I take the drugs together without any problems?
Patient	This morning the hepatologist prescribed me Glecaprevir/Pibrentasvir for a chronic HCV infection. I forgot to tell him that I'm taking atorvastatin for dyslipidemia. Can I take the drugs together without any problems?
Physician	I need to start therapy with direct‐acting antivirals in a 75‐year‐old patient recently diagnosed with HCV. I do not know his genotype. He has no evidence of advanced chronic liver disease. He is taking dabigatran for a recent pulmonary thromboembolism. What is the best treatment option considering potential drug–drug interactions?
Physician	A 55‐year‐old patient has just been diagnosed with chronic HCV infection. She needs to start antiviral therapy with direct‐acting antivirals as soon as possible. She has been taking atorvastatin for dyslipidemia which is currently under excellent control. What is the best treatment option?
**Domain #4: Treatment in patients with renal impairment**	
General	Can renal function influence the choice of treatment regimen to be used in a newly diagnosed patient with chronic HCV infection who is candidate for therapy with direct‐acting antivirals?
Patient	I have been chronically infected with HCV for about 20 years but have been advised against starting antiviral therapy because I am on haemodialysis. Should I go to a liver disease referral centre?
Physician	I should start antiviral therapy for a chronic HCV infection, genotype 1 and naive to antiviral therapy, in a non‐cirrhotic patient with severe renal impairment (eGFR < 30 mL/min/1.73m^2^). Can I use Sofosbuvir/Velpatasvir or would it be better to use Glecaprevir/Pibrentasvir?

In addition to the question set, M.G. and N.P. developed 25 simulated clinical cases (fully reported in the Data S1) to evaluate the models' performance in recommending appropriate HCV treatments. These cases were reviewed and validated by the senior hepatologist (A.A.) to ensure clinical relevance and accuracy. The clinical cases were designed to represent the diverse spectrum of patients encountered in HCV clinical practice. Each case included specific details about: patient demographics (age, sex), HCV genotype and viral load, previous treatment history (treatment‐naïve or treatment‐experienced), presence and degree of liver fibrosis/cirrhosis, renal function status, relevant comorbidities and concomitant medications with potential for drug–drug interactions.

### 2.2 Qualitative Answer Evaluation of Open‐Ended Questions

The primary aim of the study was to have HCV experts evaluate qualitatively the accuracy and clarity of LLM‐generated responses. To ensure comprehensive assessment, we recruited two distinct groups of evaluators. The first group consisted of four expert hepatologists selected from the main authors and chairs of the EASL HCV guidelines [8] (F.N., M.P., J.M.P. and X.F.), which were used as the dataset for both the RAG external knowledge and for training in SFT. The second group comprised four expert hepatologists from a tertiary hepatology referral center (Humanitas University Hospital, Milan) who had no direct role in HCV guideline development, providing an independent perspective from clinical practitioners. None of the evaluators from either group were involved in any phase of question creation, answer generation or dataset creation for fine‐tuning purposes. Evaluators, who were blinded to model configuration, reviewed the same set of responses from all configurations for each question before moving on to the next group of responses. Each expert was free to review and score the response sets at their own pace, taking as much time as needed to ensure a thorough assessment. This self‐paced approach helped mitigate potential fatigue or rush‐related biases.

#### 2.2.1 Accuracy

Given the lack of a widely accepted definition of accuracy and the absence of standardised quantification methods, as well as the use of multiple accuracy definitions in current literature, we opted to evaluate accuracy using both a binary grading system and a 10‐point Likert scale. We chose a 10‐point scale, despite other studies using 5‐ or 7‐point scales [13], because scales with < 10 points have been proven to offer greater sensitivity and the ability to detect more nuanced differences in responses, while increasing reliability, validity and data consistency [28, 29]. This dual approach enables us to distinguish not only completely correct answers from incorrect ones but also to gauge the level of inaccuracy among the incorrect responses. While the binary classification helps identify totally accurate answers, the Likert scale provides insights into the relative accuracy of all answers, which is especially important for identifying and addressing partial inaccuracies. The complete classification for the 10‐point Likert scale is reported in Table 2. For the binary scale, graders assigned a score of one for answers that were entirely correct and presented informative text without any hallucinations, and a score of zero for answers that did not meet these criteria.

**TABLE 2:** Grades and definitions of the 10‐point scale to evaluate the accuracy of answers generated by each model.

Grades	Definition
Grade: 0	Completely Inaccurate: The output is entirely irrelevant, incorrect or nonsensical, showing no understanding of the query.
Grade: 1	Extremely Inaccurate: The response contains major errors and misunderstandings, with very little relevant or accurate information.
Grade: 2	Highly Inaccurate: Significant inaccuracies dominate the response, though there might be a minor element of relevance or accuracy.
Grade: 3	Very Inaccurate: While mostly inaccurate, the response shows some basic understanding of the topic, but with major errors.
Grade: 4	Inaccurate: The response has a mix of correct and incorrect information, but the inaccuracies are more prominent.
Grade: 5	Moderately Accurate: The response is a balance of accurate and inaccurate information, showing an equal mix of correct insights and errors.
Grade: 6	Somewhat Accurate: The response is more accurate than not, with some notable inaccuracies but a general understanding of the topic.
Grade: 7	Mostly Accurate: The response contains mostly correct information, with minor errors or inaccuracies.
Grade: 8	Very Accurate: The response is highly accurate, with only very slight inaccuracies or areas of uncertainty.
Grade: 9	Highly Accurate: The response is extremely accurate, showing a deep understanding of the topic with almost no inaccuracies.
Grade: 10	Completely Accurate: The response is entirely accurate, with no discernible inaccuracies or errors, perfectly addressing the query.

#### 2.2.2 Clarity

Clarity was defined as the presence of relevant information that could be easily understood, straight to the point, explicit and free from ambiguity. In evaluating clarity, the graders provided a binary score of one for clear answers and a score of zero for unclear answers.

### 2.3 Quantitative Answer Evaluation of Open‐Ended Questions

Two junior hepatologists (M.G. and N.P.) who drafted the questions also provided free‐text answers, which were reviewed and approved by the senior hepatologist (A.A.). The expert responses were used as the reference text to develop and evaluate embedding‐based text similarity metrics (i.e., cosine similarity) to determine the best chunking strategy in the RAG framework, and in the process of hyperparameter tuning. For the embedding‐based metrics, text was first tokenised internally by OpenAI's cl100k_base tokenizer, which splits text into subword tokens using byte‐pair encoding (BPE), and then converted into its embedding representation. An embedding is a high‐dimensional vector representation of data, typically text, which captures the semantic and syntactic nuances of the input [30]. We used one of the currently available text‐embedding models provided by OpenAI (i.e., text‐embedding‐3‐large) [31], which generates embeddings with 3072 dimensions for each text input. The embedding vectors were then used to compute similarity scores between expert responses and model‐generated answers. For better visualisation of the relative gap between the cosine similarity score from different models, we provide the transformation of first normalising the similarity raw score with its maximum attainable score and then applying the logit function.

### 2.4 Quantitative Evaluation of Clinical Scenario Treatment Recommendation

For each case, a gold standard for appropriate DAA regimen selection was established through a consensus process involving four independent hepatologists from tertiary referral centers, who were not involved in case creation. Their recommendations were determined by majority vote, with subsequent expert review for cases lacking initial consensus. We used these consensus‐based gold standard recommendations to evaluate the performance of each model configuration across all 25 clinical scenarios reported in detail in Data S1. Model performance was assessed by comparing model recommendations to the expert majority consensus, using complete accuracy as the metric: ‘all recommended treatment regimens without any incorrect recommendations’. This metric assessed whether the model could identify all appropriate treatment options according to the expert consensus, without suggesting any treatments that diverged from the consensus. This represents an optimal standard for clinical decision support, as it would provide clinicians with the full range of appropriate treatment options.

### 2.5 Model Configurations and Answer Generation

Experiments were conducted on a local Python (version 3.11) environment, using the OpenAI Application Programming Interface (API) (version 1.17) to interact with GPT models using the model ‘gpt‐4‐turbo‐2024‐04‐09‐preview’ according to OpenAI's nomenclature. We deliberately chose not to evaluate advanced prompting strategies in this study, as our previous research has demonstrated that performance gains achieved through sophisticated prompting techniques are substantially less significant compared to those obtained through retrieval augmentation or fine‐tuning approaches [26, 27]. Additionally, limiting the number of experimental conditions allowed us to reduce the substantial labour burden associated with expert human grading while focusing on the most promising enhancement methods for clinical applications.

However, we developed and integrated tailored heuristic prompts to direct answer generation for each model configuration, with specific prompting strategies designed separately for the 15 open‐ended questions and the 25 clinical scenarios. These prompts were crafted to elicit relevant medical information and treatment recommendations in a clinically appropriate format, as detailed in Tables S1 and S2.

Prior to the main evaluation, we conducted a comprehensive hyperparameter optimisation process, which is essential to determine the correct hyperparameter combination that reduces the model's tendency to explore less likely tokens, thus reducing the risk of hallucinations [32, 33, 34]. Using the open‐ended questions as a test bed, we systematically explored the impact of Temperature and Top_*p* settings on response quality. For Temperature, we generated 10 responses at each threshold from 0 to 2.0 in increments of 0.2, while holding Top_*p* constant at its default value. Similarly, for Top_*p*, we generated 10 responses at thresholds from 0 to 1.0 in increments of 0.1, while maintaining Temperature at its default setting. Response quality was evaluated by measuring similarity to expert‐provided answers using embedding‐based metrics. We simultaneously optimised the RAG framework by testing three different chunking strategies across varying numbers of retrieved chunks, generating 10 responses for chunk number. This systematic evaluation allowed us to identify the optimal chunking strategy and retrieval depth. Based on these optimisation experiments, we selected the highest‐performing hyperparameter values and RAG configuration to generate the responses for expert evaluation. For qualitative grading, only the first generated response was considered, simulating real‐world application conditions. The optimised parameters were subsequently applied to generate responses for the 25 clinical scenarios, where we again conducted 10 interaction rounds per case to assess recommendation accuracy and consistency.

#### 2.5.1 Baseline Model

As the baseline, we used the model employing only the heuristic prompts detailed in the Tables S1 and S2 without any enhancement techniques.

#### 2.5.2 Retrieval Augmented Generation

For the RAG [35] framework, we chose the EASL guidelines on HCV [8] as the reference text based on our group of expert regions of origin and medical practice. The reference text was provided in an LLM‐friendly version as previously validated by manually removing non‐informative data (e.g., headers or reference numbers), and converting all non‐textual sources (e.g., graphical tables containing drug–drug interactions) to text‐based lists [27]. We explored the impact of the number of retrieved chunks across three chunking strategies [36] to optimise retrieval within the RAG framework: sentence‐based chunking, in which a special delimiter was inserted at each period to isolate individual sentences; fixed‐length chunking, which segmented the text into 512‐token units with a 100‐token overlap between adjacent chunks; and paragraph‐based chunking, in which paragraphs were manually delineated to preserve semantic coherence. Each chunk was then encoded into a 3072‐dimensional embedding vector by the embedding model using OpenAI's text‐embedding‐3‐large [31]. To optimise retrieval efficiency, we utilised the Facebook AI Similarity Search (FAISS) library for vector indexing and similarity search. The embeddings were normalised and stored as float32 numpy arrays to ensure computational efficiency. For retrieval, user queries underwent the same tokenisation and embedding process; cosine similarity scores were computed between the query embedding and each chunk embedding using FAISS, and chunks with the highest score were ranked accordingly. To generate answers, we employed the paragraph‐based chunking strategy (which performed best across all tests) with two distinct retrieval configurations: a ‘RAG‐Top1’ configuration that retrieved only the single most relevant chunk, and a ‘RAG‐Top10’ configuration that retrieved the top 10 most relevant chunks based on cosine similarity scores. While the similarity improvement from 1 to 10 chunks was not dramatically different (with both configurations providing good performance), our analysis (Figure 2) showed that performance gains reached a plateau after approximately 10 chunks, offering an optimal balance between answer quality and computational efficiency. Finally, the code for the complete RAG‐based interface is available in our GitHub Repository [37].

#### 2.5.3 Supervised Fine‐Tuning

**FIGURE 1:**
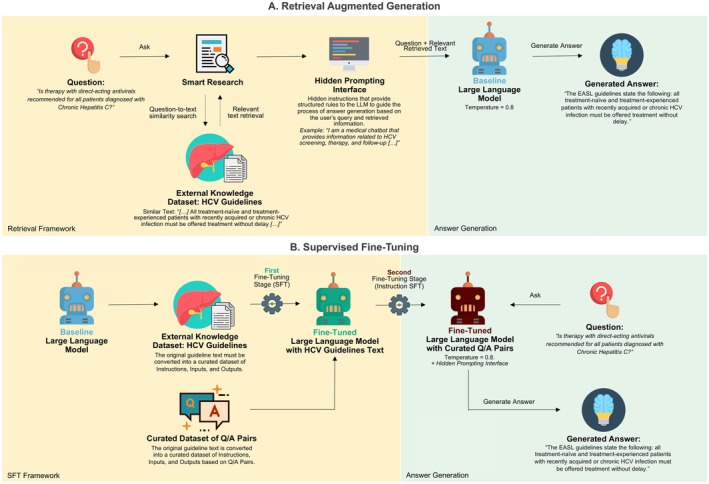
Overview of retrieval‐augmented generation and supervised fine‐tuning. In the retrieval‐augmented generation approach (Section A), a user question initiates a question‐to‐text similarity search that retrieves relevant information from external knowledge datasets, such as HCV guidelines. A hidden prompting interface then provides structured instructions to guide the large language model in generating an accurate answer based on the query and retrieved text. The baseline large language model, such as GPT‐4 Turbo, processes the question and relevant text to generate a comprehensive answer. In the supervised fine‐tuning (Section B), the process begins with a baseline large language model, which undergoes a first fine‐tuning stage using a dataset that includes HCV guidelines text. This is followed by a second fine‐tuning stage (instruction fine‐tuning) incorporating a curated dataset of Q/A pairs. The final fine‐tuned model responses are guided by hidden prompting instructions and can generate responses without searching external datasets. HCV, hepatitis C virus; SFT, supervised fine‐tuning; Q/A, question/answer.

The fine‐tuning framework was conducted on OpenAI's platform through a structured process involving two stages and four curated datasets developed according to OpenAI's instructions [38] using the *jsonl* file format. Each entry in the dataset contained a json object with fields for role, content and metadata. The training hyperparameters of batch size, learning rate multiplier and the number of epochs were set to ‘auto’ OpenAI internally ran extensive experiments on billions of tokens and hundreds of tasks during the development of InstructGPT. The default hyperparameter with the ‘auto’ set‐up is those that, on average, offer the best trade‐off between convergence speed, robustness and the risk of overfitting on heterogeneous datasets [39]. To avoid the loss of relevant information by splitting the EASL guideline dataset, we used the EASL guideline to build the training dataset and the HCV guidelines developed by the American Association of the Study of The Liver to construct the validation dataset [6]. As reported on the fine‐tuning command panel, this initial fine‐tuning phase used 723 837 tokens and showed a 0.45 training loss and 0.97 validation loss (these parameters are determined automatically after successful training and validation and reported on OpenAI's fine‐tuning platform). We further enhanced the fine‐tuned model using a curated question‐and‐answer dataset based on expert knowledge to align the model's outputs with the expertise. For this stage of Instruction Fine‐Tuning, a curated dataset was created combining the original training data with question‐answer (Q/A) pairs derived from the EASL guidelines to capture all the content contained in the guideline text in a Q/A format. A separate validation set from the American guidelines was used to evaluate model performance. As reported on the fine‐tuning command panel, the second stage used 172 887 tokens and showed a 0.01 training loss and a 1.34 validation loss (these parameters are determined automatically after successful training and validation and reported on OpenAI's fine‐tuning platform). The interpretation of these metrics remains proprietary to OpenAI and, therefore, is reported only for reproduction purposes. The Q/A pairs used for each round of instruction fine‐tuning differ from the questions used for performance evaluation, and they are available for download in our GitHub repository [37]. A summary of the SFT framework is depicted graphically in Figure 1B.

### 2.6 Statistical Analysis

For the continuous 10‐point Likert Scale, agreement among graders was evaluated using the One‐Way Random Intraclass Correlation Coefficient [40], with values reported in an interval between 0 and 1. According to Cicchetti et al. [41], we considered the following interpretations: ICC < 0.40: poor agreement; ICC ≥ 0.40 and < 0.60: fair agreement; ICC ≥ 0.60 and < 0.75: good agreement; ICC ≥ 0.75: excellent agreement. For the categorical evaluations, agreement among graders was evaluated using Fleiss' Kappa, whose values were interpreted as follows: Kappa < 0.20: slight agreement; Kappa ≥ 0.20 and < 0.40: fair agreement; Kappa ≥ 0.40 and < 0.60: moderate agreement; Kappa ≥ 0.60 and < 0.80: substantial agreement; Kappa ≥ 0.80: almost perfect or excellent agreement [42]. Data were reported as the mean and standard deviation for the 10‐point accuracy scale. We employed the chi‐squared test to compare (binary) accuracy and clarity between all configurations of experimental settings (baseline, RAG and SFT) and the paired sample Student's *t*‐test to compare differences among the 10‐point accuracy scale grading between all configurations (baseline, RAG and SFT). We employed the Mann–Whitney test to compare differences among accuracy for all configurations across all 25 clinical cases. Due to the number of multiple comparisons (*n* = 4), we applied the Bonferroni correction and considered significant a two‐tailed *p*‐value < 0.012. To conduct the analysis, we used Python v3.11 and SciPy v1.11.

## 3 Results

### 3.1 Optimal Number and Strategy of Text Chunking

**FIGURE 2:**
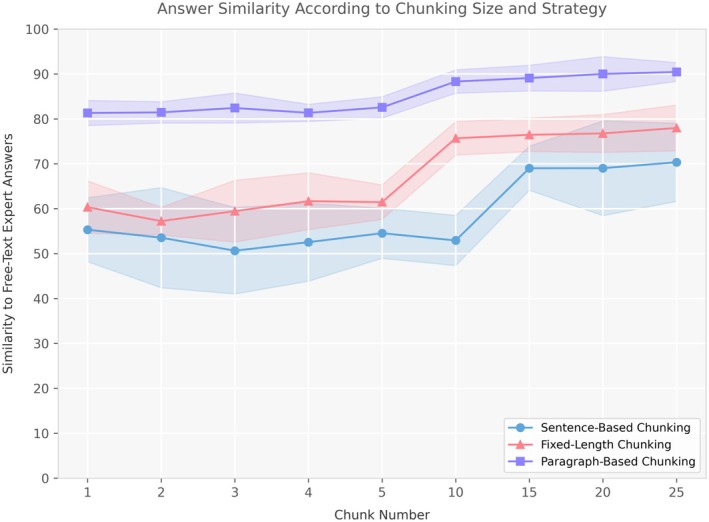
Comparison of answer similarity to expert responses across different chunking strategies and chunk numbers in the retrieval framework. Three chunking approaches were evaluated: Sentence‐based (blue), fixed‐length (red) and paragraph‐based (purple). The shaded areas around each line represent the distribution range (minimum to maximum values) of similarity scores across multiple test questions for each chunking strategy, illustrating the consistency and variability of performance at different chunk numbers.

To build the optimal RAG framework, we evaluated three chunking strategies—sentence‐based, fixed‐length and paragraph‐based—across nine different chunk counts [1, 2, 3, 4, 5, 10, 15, 19, 26] and measured similarity to free‐text expert answers (Figure 2). Paragraph‐based chunking consistently outperformed both the sentence‐based baseline (55%–70% similarity) and fixed‐length chunking (60%–78%), achieving 81%–90% similarity as the number of chunks increased. Notably, most of the gain in paragraph‐based performance occurs by retrieving the top 10 chunks, reaching a plateau thereafter. To strike the best balance between answer quality and computational cost, we therefore focused subsequent comparisons on two retrieval scenarios: Top‐1 versus Top‐10 chunk numbers using the paragraph‐based strategy. By doing so, we capture the steep initial improvement afforded by paragraph chunks while containing the overhead of larger retrieval sets.

### 3.2 Optimal Hyperparameters Tuning

**FIGURE 3:**
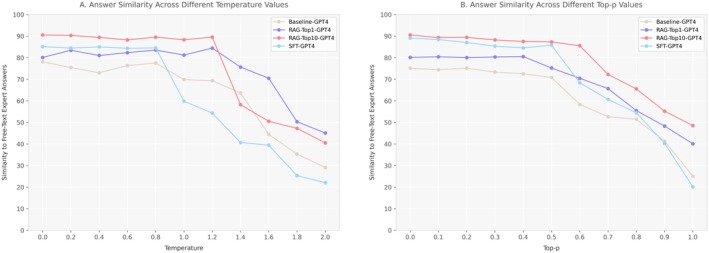
Impact of hyperparameter settings on answer similarity across different model configurations. Panel A shows the effect of temperature values (0–2.0) on similarity to expert answers, while Panel B illustrates the impact of Top‐*p* values (0–1.0).

Prior to generating responses for expert evaluation, we conducted a comprehensive hyperparameter analysis to determine the optimal settings for answer generation using cosine similarity with the free‐text answers provided by one of the guideline authors using the question sample reported in Table 1. Figure 3 illustrates the impact of temperature and Top‐*p* hyperparameters on model response similarity to expert answers. For all models (Baseline‐GPT4, RAG‐Top1‐GPT4, RAG‐Top10‐GPT4 and SFT‐GPT4), optimal performance is achieved with low values of both temperature (0–0.8) and top‐*p* (0–0.5). Notably, the RAG‐Top10‐GPT4 model maintains high similarity (> 85%) up to temperature 1.2 and top‐*p* 0.6, while other models deteriorate more rapidly. All models exhibit a dramatic performance decline with temperature ≥ 1.4 and Top‐*p* ≥ 0.7, confirming that higher values introduce excessive variability in responses. These findings suggest that to optimise accuracy in specialised question‐answering tasks, it is preferable to maintain temperature between 0 and 0.8 and Top‐*p* between 0 and 0.5, with the RAG‐Top10‐GPT4 model offering the greatest robustness to variations in these parameters. Based on these results, we selected the final values of temperature = 0.8 and Top‐*p* = 0.5 for response generation, as these represent the highest parameter values before the rapid performance decline, balancing response quality with appropriate diversity.

### 3.3 Inter‐Grader Agreement

The inter‐grader agreement between experts was evaluated using the Intraclass Correlation Coefficient (ICC) for the 10‐point scale and Fleiss' kappa for binary evaluations, as summarised in Table 3. Among guideline authors, the highest agreement was observed for RAG‐Top1 with an ICC of 0.87 (95% CI 0.76–0.94, *p* < 0.001) for 10‐point accuracy and a kappa of 0.68 (95% CI 0.45–0.88, *p* < 0.001) for binary accuracy, while RAG‐Top10 showed the highest clarity agreement with a kappa of 0.49 (95% CI 0.30–0.82, *p* = 0.031). Among tertiary referral centre experts, RAG‐Top10 demonstrated the highest agreement across all metrics with an ICC of 0.72 (95% CI 0.56–0.82, *p* < 0.001) for 10‐point accuracy and kappa values of 0.51 (95% CI 0.19–0.78, *p* < 0.001) and 0.59 (95% CI 0.10–0.92, *p* < 0.001) for binary accuracy and clarity, respectively. For complete results across all configurations, see Table 3.

**TABLE 3:** Inter‐grader agreement was calculated using the Intraclass Correlation Coefficient (ICC) for the 10‐Point Likert Scale and using Fleiss' Kappa for the binary evaluation of accuracy and clarity.

Configuration	10‐point scale accuracy grading		Binary accuracy grading		Binary clarity grading	
	ICC (95% CI)	Significance	Kappa (95% CI)	Significance	Kappa (95% CI)	Significance
**Expert Hepatologists (Guideline Authors)**						
Baseline	0.76 (0.49–0.91)	*p* < 0.001	0.30 (0.09–0.50)	*p* = 0.049	0.10 (−0.13–0.45)	NS
RAG‐Top1	0.87 (0.76–0.94)	*p* < 0.001	0.68 (0.45–0.88)	*p* < 0.001	0.42 (0.16–0.67)	*p* = 0.002
RAG‐Top10	0.83 (0.61–0.93)	*p* < 0.001	0.41 (0.20–0.65)	*p* = 0.01	0.49 (0.30–0.82)	*p* = 0.031
SFT	0.80 (0.70–0.90)	*p* < 0.001	0.39 (0.21–0.50)	*p* = 0.042	0.41 (0.20–0.59)	*p* = 0.032
**Expert Hepatologists (Tertiary Referral Center)**						
Baseline	0.24 (0.10–0.47)	*p* = 0.004	0.24 (−0.04–0.48)	NS	0.12 (−0.16–0.33)	NS
RAG‐Top1	0.41 (0.10–0.71)	*p* < 0.001	0.43 (0.10–0.67)	*p* = 0.033	0.45 (0.30–0.60)	*p* = 0.026
RAG‐Top10	0.72 (0.56–0.82)	*p* < 0.001	0.51 (0.19–0.78)	*p* < 0.001	0.59 (0.10–0.92)	*p* < 0.001
SFT	0.47 (0.22–0.65)	*p* < 0.001	0.41 (0.22–0.69)	*p* = 0.037	0.45 (0.10–0.60)	*p* = 0.031

Abbreviations: 95% CI, 95% confidence interval; NS, not significant.

### 3.4 Evaluation of Accuracy and Clarity by Human Graders

The comparative performance in accuracy and clarity grading between the baseline GPT‐4 Turbo, RAG‐Top1, RAG‐Top10 and SFT configurations is summarised in Table 4. Among guideline authors, RAG‐Top10 showed the highest mean accuracy of 9.45 (±1.42), significantly higher (*p* < 0.001) than the baseline GPT‐4 Turbo's 6.4 (±2.30). RAG‐Top1 achieved 8.1 (±3.13), while the SFT model performed well with 8.3 (±2.03), both significantly outperforming the baseline (*p* = 0.001 and *p* < 0.001 respectively). In binary accuracy grading, RAG‐Top10 showed the highest performance (91.7% accuracy), followed by RAG‐Top1 (81.7%) and SFT (71.7%), all significantly outperforming the baseline (36.6%, *p* < 0.001). Regarding clarity, RAG‐Top10 (91.7%), RAG‐Top1 (86.6%) and SFT (88.3%) all significantly outperformed the baseline (46.6%, *p* < 0.001). Among tertiary referral centre experts, RAG‐Top10 demonstrated the best performance with a mean accuracy of 8.40 (±0.99), significantly higher than baseline's 7.23 (±1.66, *p* < 0.001) and RAG‐Top1's 7.38 (±1.42, *p* < 0.001), with no significant difference from SFT's 8.25 (±1.43). For binary accuracy, RAG‐Top10 achieved the highest rating (93.3%), significantly outperforming baseline (50%, *p* < 0.001), RAG‐Top1 (60%, *p* < 0.001) and SFT (76.7%, *p* = 0.010). In clarity evaluations, RAG‐Top10 again led with 96.7%, significantly better than baseline (65%, *p* < 0.001) and RAG‐Top1 (75%, *p* < 0.001), with no significant difference from SFT (83.3%).

**TABLE 4:** Comparisons of accuracy and clarity grading between the baseline model and the RAG and SFT model pipelines.

Configuration	10‐point scale accuracy grading		Binary accuracy grading		Binary clarity grading	
	Mean (±SD)	Significance	Number (%)	Significance	Number (%)	Significance
**Expert Hepatologists (Guideline Authors)**						
Baseline	6.4 (±2.30)	vs. RAG‐Top1: *p* = 0.001 vs. RAG‐Top10: *p* < 0.001 vs. SFT: *p* < 0.001	22 (36.6%)	vs. RAG‐Top1: *p* < 0.001 vs. RAG‐Top10: *p* < 0.001 vs. SFT: *p* < 0.001	28 (46.6%)	vs. RAG‐Top1: *p* < 0.001 vs. RAG‐Top10: *p* < 0.001 vs. SFT: *p* < 0.001
RAG‐Top1	8.1 (±3.13)	vs. Baseline: *p* = 0.001 vs. RAG‐Top1: *p* = 0.005 vs. SFT: NS	49 (81.7%)	vs. Baseline: *p* < 0.001 vs. RAG‐Top1: NS vs. SFT: NS	52 (86.6%)	vs. Baseline: *p* < 0.001 vs. RAG‐Top10: NS vs. SFT: NS
RAG‐Top10	9.45 (±1.42)	vs. Baseline: *p* < 0.001 vs. RAG‐Top1: *p* = 0.005 vs. SFT: *p* < 0.001	55 (91.7%)	vs. Baseline: *p* < 0.001 vs. RAG‐Top1: NS vs. SFT: NS	55 (91.7%)	vs. Baseline: *p* < 0.001 vs. RAG‐Top1: NS vs. SFT: NS
SFT	8.3 (±2.03)	vs. Baseline: *p* < 0.001 vs. RAG‐Top1: NS vs. RAG‐Top10: *p* < 0.001	43 (71.7%)	vs. Baseline: *p* < 0.001 vs. RAG‐Top1: NS vs. RAG‐Top10: NS	53 (88.3%)	vs. Baseline: *p* < 0.001 vs. RAG‐Top1: NS vs. RAG‐Top10: NS
**Expert Hepatologists (Tertiary Referral Center)**						
Baseline	7.23 (±1.66)	vs. RAG‐Top1: NS vs. RAG‐Top10: *p* < 0.001 vs. SFT: *p* < 0.001	30 (50%)	vs. RAG‐Top1: NS vs. RAG‐Top10: *p* < 0.001 vs. SFT: *p* = 0.002	39 (65%)	vs. RAG‐Top1: NS vs. RAG‐Top10: *p* < 0.001 vs. SFT: NS
RAG‐Top1	7.38 (±1.42)	vs. Baseline: NS vs. RAG‐Top10: *p* < 0.001 vs. SFT: *p* = 0.001	36 (60%)	vs. Baseline: NS vs. RAG‐Top10: *p* < 0.001 vs. SFT: NS	45 (75%)	vs. Baseline: NS vs. RAG‐Top10: *p* < 0.001 vs. SFT: NS
RAG‐Top10	8.40 (±0.99)	vs. Baseline: *p* < 0.001 vs. RAG‐Top1: *p* < 0.001 vs. SFT: NS	56 (93.3%)	vs. Baseline: *p* < 0.001 vs. RAG‐Top1: *p* < 0.001 vs. SFT: *p* = 0.010	58 (96.7%)	vs. Baseline: *p* < 0.001 vs. RAG‐Top1: *p* < 0.001 vs. SFT: NS
SFT	8.25 (±1.43)	vs. Baseline: *p* < 0.001 vs. RAG‐Top1: *p* = 0.001 vs. RAG‐Top10: NS	46 (76.7%)	vs. Baseline: *p* = 0.002 vs. RAG‐Top1: NS vs. RAG‐Top10: *p* = 0.010	50 (83.3%)	vs. Baseline: NS vs. RAG‐Top1: NS vs. RAG‐Top10: NS

*Note:* Statistical testing is based on pairwise comparison (paired Student's *T*‐test for continuous variables and chi‐squared test for binary variables). According to the Bonferroni correction, a *p*‐value < 0.012 can be considered statistically significant.

Abbreviations: RAG, Retrieval Augmented Generation; SFT, Supervised Fine‐Tuning.

### 3.5 Model Performance on Simulated Clinical Scenarios

To establish a gold standard for direct‐acting antivirals (DAAs) regimen selection, recommendations were determined by majority vote among four hepatologists from tertiary referral centres across 25 simulated clinical scenarios. Expert consensus was achieved for 25 (100%) sofosbuvir‐alone recommendations, 20 (80%) sofosbuvir‐velpatasvir recommendations, 23 (92%) glecaprevir‐pibrentasvir recommendations, 20 (80%) grazoprevir‐elbasvir recommendations and all 25 (100%) decisions regarding ribavirin addition. For cases lacking initial majority consensus, subsequent expert review yielded final gold standard prescribing patterns: sofosbuvir‐alone in 0 (0%) cases, sofosbuvir‐velpatasvir in 23 (92%) cases, sofosbuvir‐velpatasvir‐voxilaprevir in 7 (28%) cases, glecaprevir‐pibrentasvir in 18 (72%) cases, grazoprevir‐elbasvir in 3 (12%) cases and ribavirin in 0 (0%) cases. We evaluated various model configurations by conducting 10 independent interactive sessions per configuration across all 25 clinical scenarios, using identical prompts throughout. Model performance was assessed using median and interquartile ranges (IQR). The baseline model demonstrated a median accuracy of 24.0% (IQR: 22%), the RAG‐Top1 configuration showed marked improvement with a median accuracy of 58.0% (IQR: 10%, vs. baseline, *p* < 0.001), the RAG‐Top10 configuration emerged as the superior performer, achieving a median accuracy of 76.0% (IQR: 8%, vs. baseline, *p* < 0.001) and the SFT model demonstrated a median accuracy of 66.0% (IQR: 19%, vs. baseline, *p* < 0.001).

## 4 Discussion

This is one of the first studies in the literature to investigate the potential of SFT for answer generation and grading by multiple guideline panel experts in the field of digestive diseases [21, 25]. Both the RAG and SFT frameworks outperform the baseline model in terms of accuracy and clarity for questions related to HCV management. In addition, the use of binary metrics for evaluating accuracy and clarity resulted in a slight to fair agreement among graders, in contrast to using a point‐based scale, which resulted in a higher agreement.

### 4.1 Hyperparameter Optimization and Best Chunking Strategy

Our hyperparameter optimization findings align remarkably well with those reported in a recent study that evaluated 27 LLM configurations [21]. We similarly identified optimal temperature values between 0 and 0.8 and top‐*p* values between 0 and 0.5. Our study confirms that paragraph‐based chunking significantly outperforms both sentence‐based and fixed‐length strategies, achieving 81%–90% similarity to expert answers [21]. This superiority stems from paragraphs preserving semantic context and thematic coherence while providing an optimal information unit size for LLM processing. Additionally, we observed that most performance gains occur within the first 10 retrieved chunks, after which returns diminish—a threshold that mirrors recently published optimal configurations [43, 44]. This convergence of results across independent studies strengthens confidence in these hyperparameter settings for clinical LLM applications, particularly in hepatology, where guideline adherence is critical for patient safety.

### 4.2 RAG and SFT Increase Accuracy of LLM‐Generated Text

Our study suggests significant improvements in accuracy and clarity when utilising RAG and SFT frameworks when generating answers for open‐ended questions. Our RAG‐Top10 framework was the most accurate model for both guideline experts (average 10‐point scale: 9.45, binary evaluation: 91.7%) and hepatologists from the tertiary referral centre (average 10‐point scale: 8.40, binary evaluation: 93.3%). Similarly, SFT improved performance with mean accuracy scores for the two groups of evaluators when compared to baseline. This confirms that incorporating domain knowledge through RAG with relevant guidelines reduces hallucinations (i.e., plausible sounding but incorrect responses) and training bias (absence of relevant information in the training knowledge) [15, 16, 42, 43].

In terms of accuracy of the baseline GPT‐4 model, similar performance was demonstrated with larger question datasets on colorectal cancer, inflammatory bowel disease and irritable bowel syndrome [45]. Other studies have reported higher accuracy metrics, up to 91.4%, when GPT‐4 Turbo was queried about common patients' requests on colonoscopy or follow‐up after polypectomy [46, 47]. However, when RAG‐enhanced GPT‐4 was compared to foundational GPT‐4 for predicting adequate follow‐up recommendations after polypectomy, accuracy increased from 50.5% to 79%, similar to our study [26]. In contrast, our previous exploration using a framework for retrieving information from guidelines achieved an accuracy of 99% [27]. However, this could indicate a reduction in accuracy performance compared to the method itself. This methodological framework prioritised the fidelity of the extracted information from the external text dataset. Each question was directed toward a specific part of the text, avoiding nuances of significance and the need for interpretation as in the case of this manuscript, and we lacked world experts as both question designers and graders.

Interestingly, when world experts graded the responses generated by the LLMs, there was a notable gap in inter‐rater agreement across different configurations. Specifically, the Fleiss' Kappa for binary accuracy for the RAG‐Top10 was 0.41 according to the guideline experts and 0.51 for the tertiary referral center hepatologists. By contrast, the 10‐point Likert scale showed good to excellent agreement, with an ICC of 0.83 (guideline experts) and 0.72 (tertiary referral center hepatologists). This pattern suggests that the apparent disagreement in binary ratings may reflect the inherent limitation of forced binary choices in capturing the nuanced quality of LLM responses rather than fundamental inconsistency in expert judgement. The substantially higher agreement on Likert scales indicates that experts can reliably assess response quality when provided with appropriately granular rating options. These findings have important implications for LLM validation methodology: binary accuracy metrics, while seemingly objective, may paradoxically introduce measurement error by forcing experts to collapse complex clinical judgement into oversimplified categories. The variability observed in this study underscores the challenges in aligning human‐perceived accuracy with standardised evaluation metrics. It also highlights the need for improved methodologies that can better accommodate the nuances of LLM performance, especially in high‐stakes fields like clinical medicine.

### 4.3 RAG and SFT Increases Perceived Clarity of LLM‐Generated Text

We also measured the impact of RAG and SFT with HCV guidelines on clarity (e.g., ‘straight to the point’, ‘explicit’ and ‘free from ambiguity’). In particular, RAG‐Top10 achieved the highest clarity, with clear answers in 91.7% of cases, compared to 86.6% for RAG‐Top1, 88.3% for SFT and only 46.6% for the baseline GPT‐4 Turbo model. A similar trend was found with a second, independent panel of tertiary‐referral hepatologists. The addition of RAG or SFT, which bounds responses to a specific knowledge dataset, was shown to refine the model output, providing answers that are not only more accurate, but also more concise and targeted. The decision to use a binary evaluation for clarity, rather than a scale as employed for accuracy, was based on several considerations. Firstly, clarity is a more qualitative and subjective measure compared to accuracy. While accuracy can be more easily quantified on a scale, clarity often presents as an either/or proposition—an answer is either clear or it is not, with less room for gradation. Unlike accuracy, where nuances in correctness can be meaningful, clarity benefits from a more straightforward assessment.

### 4.4 RAG and SFT Increases Accuracy in LLM‐Recommended Treatment

We evaluated LLM performance on selecting the appropriate DAAs across 25 clinical scenarios. The RAG‐Top10 configuration prescribed all recommended treatment regimens without any incorrect recommendations in 76% of cases (vs. baseline: 24%), whereas SFT yielded lower rates of accuracy (i.e., 66%). Similar results were reported in a recent study of multiple LLMs in the context of upper gastrointestinal bleeding, where SFT‐GPT4 slightly outperformed the RAG configuration (though the difference was not statistically significant) in answering open‐ended questions, whereas the RAG configuration demonstrated clear superiority when handling the less‐structured prompts derived from clinical simulation exercises [21].

### 4.5 Strengths and Limitations

Strengths of the study include the novel exploration of an SFT framework and RAG‐enhanced LLMs for HCV management and the use of European Guidelines influence and its effects on response quality. The involvement of world experts in HCV as evaluators adds a significant layer of credibility to the accuracy estimates of these LLM enhancements. Having guideline experts as both graders and answer designers ensures we approach the gold standard in defining the accuracy of outputs, a feature not yet presented in the literature. We note that only the default settings available through the OpenAI API were utilised, which may be suboptimal for fine‐tuning on clinical guidelines. Our study serves as an initial exploratory effort in this domain, laying the groundwork and baseline expected performance that could be improved with more sophisticated frameworks. Furthermore, it aims to uncover the problem of robust evaluation methodologies. The involvement of experts as evaluators is particularly crucial due to the lack of established benchmarks for evaluating responses generated by LLMs, which could delay their safe deployment in clinical practice. In addition, most of the previous studies [13] on LLM applications in digestive diseases failed to provide transparent information regarding model specifications (e.g., version, hyperparameters), prompt engineering frameworks, precise definition of accuracy measurement and did not involve guideline experts as graders. Our study is the first in evaluating LLMs fine‐tuned on a specific domain knowledge set for generating medical advice, as opposed to the existing studies that evaluate the performance of fine‐tuned models for note summarization [48, 49].

Limitations include a narrow problem‐based focus, persistent gaps in accuracy and the representativeness of the questions to real‐world clinical care. The focus on HCV may limit the generalisability of the findings to other diseases or healthcare issues. Furthermore, although using RAG reduces the incidence of hallucinations, it does not eliminate the risk, and the lack of established benchmarks for LLM outputs remains a challenge. In addition, we only used one medical guideline in a forward pass for each new query in the RAG framework. This may affect accuracy in the case of multiple guidelines that exceed the LLM context window, leading to a chunking strategy that may affect context retrieval from the external dataset [50].

Our evaluation dataset comprised 40 distinct queries (15 open‐ended questions and 25 clinical scenarios), which, while consistent with sample sizes reported in previous LLM evaluation studies in gastroenterology [13], may be insufficient for broad clinical deployment of these tools, even within a single disease area. This limitation underscores the critical absence of standardised, publicly available benchmarks developed by relevant scientific societies for comprehensive clinical validation of LLMs. Without such established evaluation frameworks, individual studies remain constrained by resource limitations and may not capture the full spectrum of clinical complexity required for safe healthcare integration.

Another limitation is that we only evaluated GPT models, excluding other models such as open‐source alternatives. However, we have demonstrated previously the increased performance in accuracy of proprietary models when compared to open‐source models such as Meta's Llama‐2 across multiple model configurations [21]. This focus is due to the current availability of OpenAI within hospital systems. Also, we did not implement reinforcement learning from human feedback (RLHF) in our current study due to limitations on expertise and cost. RLHF represents the pivotal force driving the enhancement of LLMs and ensuring their alignment with human expectations. We are actively exploring the possibility of applying effective RLHF implementation as an area of future research.

## 5 Conclusion

The study shows that baseline LLMs have suboptimal accuracy and require approaches to inject domain‐specific expertise to improve accuracy and perceived clarity. RAG or SFT frameworks provide more accurate and clear responses than the baseline model, especially when grounded in authoritative guidelines for HCV management. Development and deployment costs can limit real‐life implementation, and generalisability remains an issue due to privacy concerns and absence of representative databases of real‐world questions.

## Author Contributions

M.G., N.P., A.A. and D.L.S. conceived and designed the analysis. M.G. and N.P. collected the data and grading provided by the experts (F.N., M.P., X.F., J.‐M.P.). M.G., S.K., M.A. and N.P. performed the analysis. All authors were involved in writing, editing and approving the final version of the manuscript.

## Conflicts of Interest

F.N.: Advisor for Gilead Sciences, IQVIA, AbbVie, Medicine Patents Pool, International Agency for Research on Cancer; Speaker for Gilead Sciences, Roche Diagnostics; Travel Grant from Gilead Sciences. A.A.: Advisory board fees from: Gilead, MSD, Abbvie, Ipsen; Speaker's fees from: Gilead, Abbvie, Ipsen. X.F.: acted as advisor for Gilead. The other authors declare no conflicts of interest with the present work. M.P.: Advisory Board/Speaker Bureau/Travel grants for Gilead Sciences, Abbvie, Merck, ViiV, GSK, Astra Zeneca, Infectofos, Pfizer, Menarini, Angelini. J.‐M.P.: advisor or speaker for Abbott, Abbvie, Gilead and GSK. None of the authors received consultancy fees from OpenAI.

## Supporting information


**Data S1:** liv70349‐sup‐0001‐Supinfo.docx.

## Data Availability

The data that support the findings of this study are available from the corresponding author upon reasonable request.
